# The interferon-related developmental regulator 1 is used by human papillomavirus to suppress NFκB activation

**DOI:** 10.1038/ncomms7537

**Published:** 2015-03-13

**Authors:** Bart Tummers, Renske Goedemans, Laetitia P. L. Pelascini, Ekaterina S. Jordanova, Edith M. G. van Esch, Craig Meyers, Cornelis J. M. Melief, Judith M. Boer, Sjoerd H. van der Burg

**Affiliations:** 1 Department of Clinical Oncology, Leiden University Medical Center, Albinusdreef 2, 2333ZA Leiden, The Netherlands; 2 Department of Molecular Cell Biology, Leiden University Medical Center, Albinusdreef 2, 2333ZA Leiden, The Netherlands; 3 Center for Gynaecological Oncology, Plesmanlaan 121, 1066CX Amsterdam, The Netherlands; 4 Department of Gynaecology, Leiden University Medical Center, Albinusdreef 2, 2333ZA Leiden, The Netherlands; 5 Department of Microbiology and Immunology, The Pennsylvania State University College of Medicine, 500 University Drive, Hershey, Pennsylvania 17033, USA; 6 Department of Immunohematology and Blood Transfusion, Leiden University Medical Center, Albinusdreef 2, 2333ZA Leiden, The Netherlands; 7 Department of Human Genetics, Leiden University Medical Center, Albinusdreef 2, 2333ZA Leiden, The Netherlands

## Abstract

High-risk human papillomaviruses (hrHPVs) infect keratinocytes and successfully evade host immunity despite the fact that keratinocytes are well equipped to respond to innate and adaptive immune signals. Using non-infected and freshly established or persistent hrHPV-infected keratinocytes we show that hrHPV impairs the acetylation of NFκB/RelA K310 in keratinocytes. As a consequence, keratinocytes display a decreased pro-inflammatory cytokine production and immune cell attraction in response to stimuli of the innate or adaptive immune pathways. HPV accomplishes this by augmenting the expression of interferon-related developmental regulator 1 (IFRD1) in an EGFR-dependent manner. Restoration of NFκB/RelA acetylation by IFRD1 shRNA, cetuximab treatment or the HDAC1/3 inhibitor entinostat increases basal and induced cytokine expression. Similar observations are made in IFRD1-overexpressing HPV-induced cancer cells. Thus, our study reveals an EGFR–IFRD1-mediated viral immune evasion mechanism, which can also be exploited by cancer cells.

## Abstract (web summary)

Human papillomavirus employs immune evasion strategies to establish a long-term infection. Here the authors show that the virus in the EGFR-dependent manner induces IFRD1, which blocks NFκB activating acetylation, and that this process can be suppressed by the EGFR inhibitor cetuximab.

## Graphical abstract

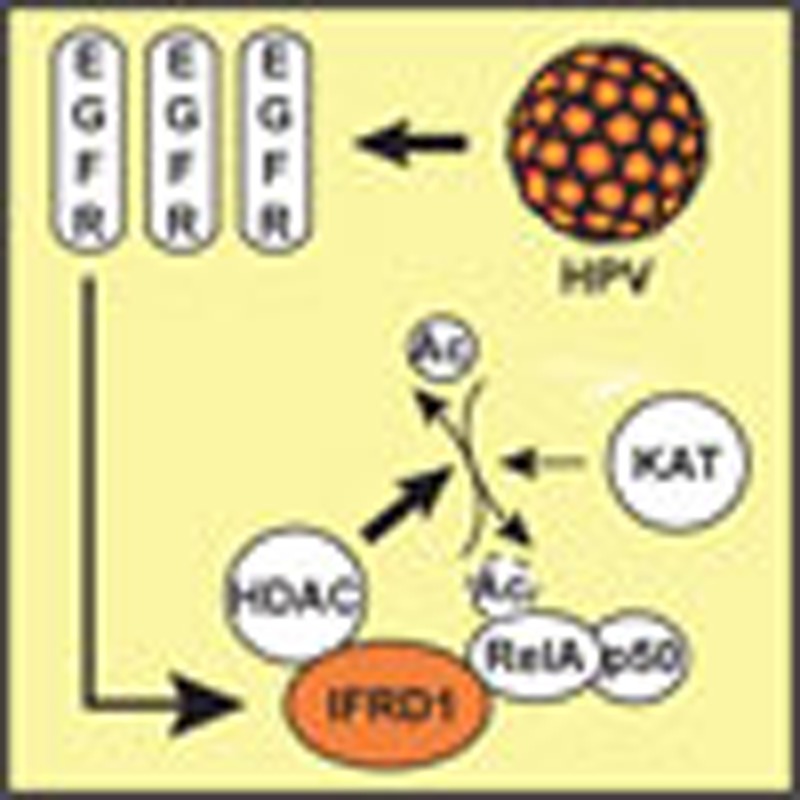

High-risk human papillomaviruses (hrHPVs) are absolutely species-specific small double-stranded DNA viruses that primarily target undifferentiated keratinocytes (KCs) of squamous epithelia via micro-wounds and abrasions. HrHPV infections can last up to 2 years despite viral activity in infected KCs, the expression of viral antigens and the presence of KC-expressed pattern recognition receptors (PRRs)^1^,^2^,^3^,^4^ that should lead to activation of innate and adaptive immune responses. This indicates that hrHPV has evolved mechanisms to transiently evade innate and adaptive immune mechanisms. Ultimately, the majority of hrHPV infections are controlled by the immune system, in particular by type-1 interferon (IFN)-γ and tumour necrosis factor (TNF)-α cytokine-producing T cells^5^. In case of immune failure, hrHPV causes cancer of the anogenital and/or head and neck regions^6^.

Upon infection, hrHPV alters the immune-related response of KCs to various innate and adaptive immune stimuli, resulting in impaired expression of IFN-stimulated genes, interferon regulatory transcription factor-induced genes and NFκB-induced genes^3^,^7^,^8^,^9^,^10^,^11^,^12^, suggesting that HPV hampers STAT1 and NFκB activation. HPV-infected KCs display downregulated basal expression of *STAT1* and lowered STAT1 protein levels, explaining the impaired expression of IFN-stimulated genes^13^,^14^,^15^,^16^. Furthermore, soon after infection HPV upregulates the cellular deubiquitinase ubiquitin carboxy-terminal hydrolase L1 (UCHL1) to impair PRR-induced NFκB activation by upstream interference with TRAF3, TRAF6 and NEMO^8^. The upregulation of UCHL1, however, cannot explain how the virus manages to suppress the KCs response to adaptive immune signals^12^. In addition, repressing UCHL1 does not fully restore NFκB signalling via PRR^8^, suggesting that one or more additional mechanisms are in play to suppress NFκB signalling.

In this study, we analyse NFκB activation and subsequent cytokine/chemokine production following IFN-γ and TNF-α stimulation in uninfected and HPV-infected primary KCs. Our study reveals that RelA acetylation, needed for NFκB transcriptional activity^17^, is impaired in hrHPV-infected KCs. The HPV-induced overexpression of the cellular protein interferon-related developmental regulator 1 (IFRD1) is shown to be instrumental in this process and involves histone deacetylases (HDACs) 1 and/or 3. The augmented expression of IFRD1 is the result of the HPV-mediated upregulation of the epidermal growth factor receptor (EGFR). Blocking of IFRD1 protein expression by small hairpin RNA (shRNA) or via the anti-EGFR antibody cetuximab restores NFκB/RelA-mediated cytokine expression. Additional data suggest that IFRD1 may have a similar role in suppressing cytokine/chemokine production in HPV-positive cervical cancer cells.

## Results

### HrHPV impairs the KCs cytokine response to IFN-γ and TNF-α

To evaluate whether the KCs immune response following the exposure to IFN-γ and/or TNF-α is attenuated by hrHPV, we utilized a system that resembles the natural infection with hrHPV as closely as possible. Primary KCs stably maintaining the hrHPV genome as episomes (hrHPV+KCs) display similar growth properties as non-transfected KCs, and upon culture in organotypic raft cultures, mimic HPV infection *in vivo* as documented by genome amplification, late gene expression and virus production during the differentiation-dependent life cycle of HPV^18^,^19^,^20^.

**Figure 1:**
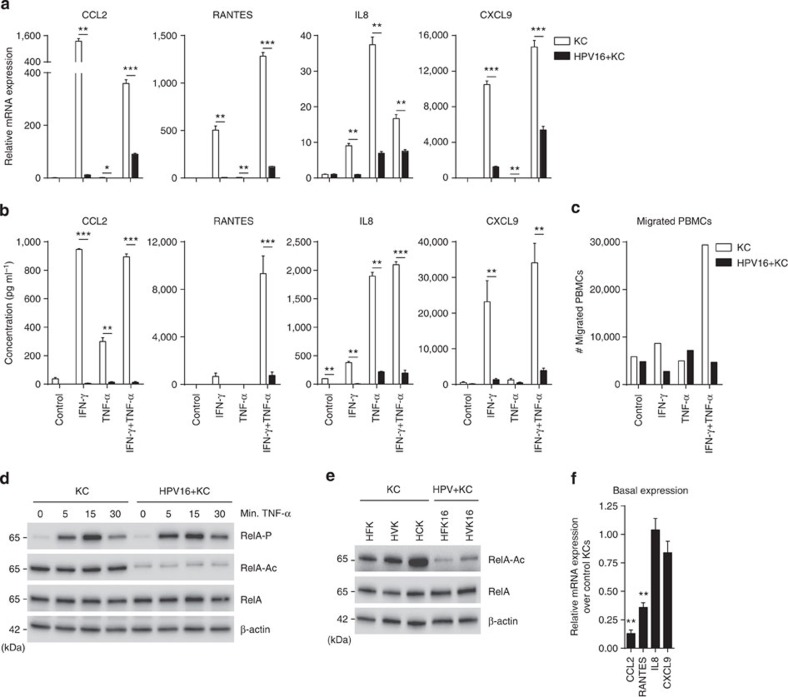
HPV16 impairs IFN-γ and TNF-α-induced cytokine production and RelA K310 acetylation in KCs. (**a**) RT–qPCR of *CCL2*, *RANTES*, *IL-8* and *CXCL9* expression by 24-h control, IFN-γ- and/or TNF-α-stimulated undifferentiated KCs or HPV16+KCs. Gene expression was normalized using *GAPDH* as the calibrator gene. Fold changes over control-stimulated undifferentiated KCs were calculated and depicted. (**b**) Enzyme-linked immunosorbent assay for CCL2, RANTES, IL-8 and CXCL9 in cleared supernatants of 24-h control, IFN-γ- and/or TNF-α-stimulated undifferentiated KCs or HPV16+KCs. (**c**) PBMCs migration towards cleared supernatants of 24-h control, IFN-γ- and/or TNF-α-stimulated KCs or HPV16+KCs. A representative example of three different donors is shown. (**d**) RelA phosphorylation, acetylation and total levels in KCs and HPV16+KCs stimulated with TNF-α for 0, 5, 15 and 30 min. (**e**) RelA acetylation and total levels at steady state in three human primary KC donor pools originating from human foreskin keratinocytes (HFKs), human vaginal keratinocytes (HVKs) or human cervical keratinocytes (HCKs) and two HPV16+genome-transfected primary KC pools of foreskin (HFK16) or vaginal (HVK16) origin. (**f**) RT–qPCR of *CCL2, RANTES, IL-8* and *CXCL9* in HPV16+KCs and KCs. Gene expression was normalized using *GAPDH* as the calibrator gene. Gene expression in HPV16+KCs was standardized over KCs. All data are representative for at least three independent experiments. Error bars indicate s.d. *P* values were determined using Welch-corrected unpaired *t*-tests. *P<0.05, **P<0.01 and ***P<0.001.

The presence of HPV type 16 (HPV16) was clearly associated with an impaired capacity to respond to IFN-γ and to TNF-α, as shown by the lower messenger RNA (mRNA) expression and production of the IFN-γ and/or TNF-α-induced pro-inflammatory cytokines CCL2, RANTES (CCL5), interleukin (IL)-8 and the chemokines CXCL9, 10 and 11 by KCs (Fig. 1a,b). Not only did the presence of HPV16 impair the production of cytokines, also the migration of peripheral blood mononuclear cells (PBMCs) to supernatants of IFN-γ and TNF-α-stimulated HPV16+KCs was greatly impaired (Fig. 1c).

These data suggest that hrHPV, besides impairing the innate immune response of KCs^8^, also suppresses the KCs response to the adaptive immune signals provided by IFN-γ and TNF-α.

The hrHPV-mediated deregulated expression of STAT1 (refs 13, 14, 15, 16) may explain the impaired cytokine expression by hrHPV-positive KCs upon IFN-γ stimulation, but not the impaired response to TNF-α (IL-8) or to IFN-γ and TNF-α (RANTES). Previously, we showed that hrHPV hampers phosphorylation of the NFκB subunit RelA (p65) upon stimulation with the innate PRR ligand poly(I:C)^8^. As TNF-α stimulation rapidly induces the phosphorylation of RelA^17^, we tested whether hrHPV also hampers rapid TNF-α-induced RelA phosphorylation by stimulating KCs and HPV16+KCs for 0, 5, 15 or 30 min with TNF-α. Western blotting showed that RelA was rapidly phosphorylated similarly in KCs and HPV16+KCs, peaking after 15 min of TNF-α stimulation (Fig. 1d), indicating that the impairment of TNF-α-induced responses seen in HPV16+KCs was not due to altered RelA phosphorylation after short-term TNF-α stimulation. Activated NFκB translocates to the nucleus where it is modified to regulate its DNA-binding ability and transcriptional activity. Acetylation of the RelA subunit at lysine 310 (K310) is crucial in this process^17^. Strikingly, acetylated RelA K310 protein levels were lower in the HPV16+KCs than in uninfected KCs, both in the absence of stimulation and after short-term TNF-α stimulation (Fig. 1d). The lowered basal RelA K310 acetylation state was verified in three independent primary KC and two independent HPV16+KC cultures (Fig. 1e), indicating that HPV hampers the activity of NFκB already at steady-state levels. This was also reflected in a lowered basal cytokine gene expression in unstimulated HPV16+KCs (Fig. 1f).

### HrHPV upregulates IFRD1 to impair RelA K310 acetylation

**Figure 2:**
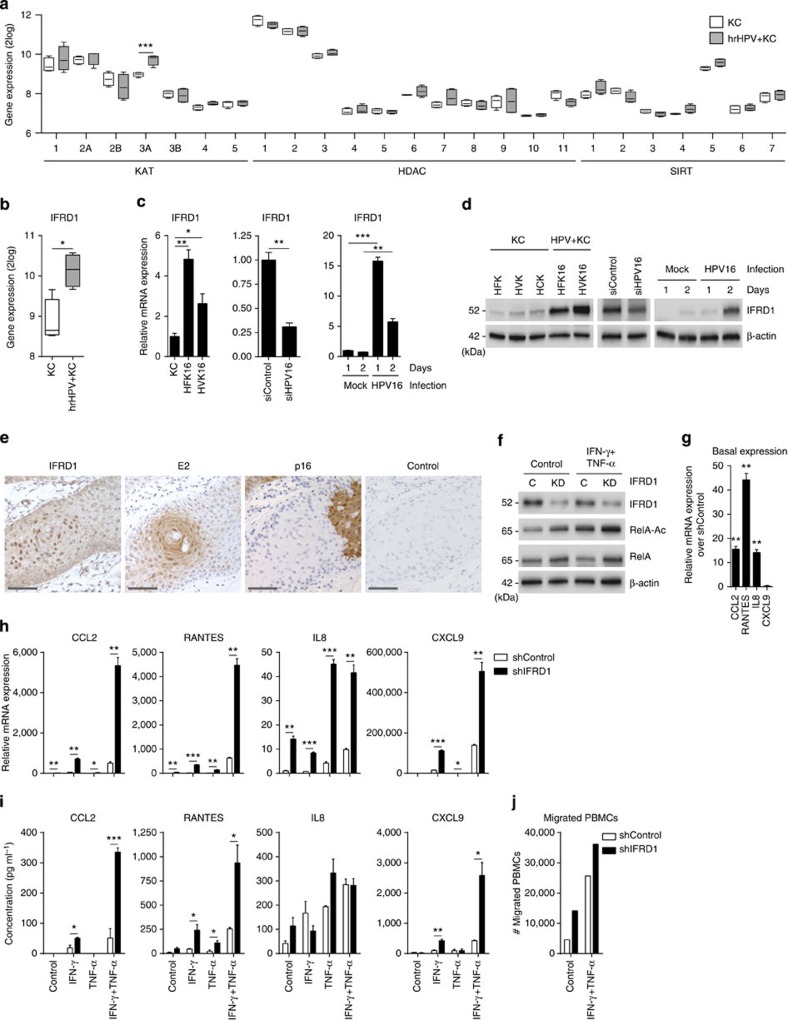
HrHPV upregulates IFRD1 to impair RelA K310 acetylation and basal cytokine expression. Microarray intensities for (**a**) all *KATs*, *HDACs* and *SIRTs*, and (**b**) *IFRD1* in four independent KCs and four independent hrHPV+KCs represented in a box plot. The box contains the 1st quartile up to the 3rd quartile; the median is represented as a line; whiskers represent the values of the outer two quartiles. (**c**) *IFRD1* mRNA expression of one representative control primary KC culture and two HPV16+KC cultures (left panel), in HFK16 cells transfected with siControl or siHPV16 (middle panel) and in primary KCs that are either mock infected or infected with native HPV16 virions (right panel), as measured by RT–qPCR. (**d**) IFRD1 protein expression in three human primary keratinocyte (KC) donor pools originating from human foreskin keratinocytes (HFKs), human vaginal keratinocytes (HVKs) or human cervical keratinocytes (HCKs) and two HPV16+genome-transfected primary KC pools of foreskin (HFK16) or vaginal (HVK16) origin (left panel) in HFK16 cells transfected with siControl or siHPV16 (middle panel) and in primary KCs that are either mock infected or infected with native HPV16 virions (right panel), as measured by western blot. (**e**) Immunohistochemical staining for IFRD1, HPV16 E2, p16 and negative antibody control of a vulvar intraepithelial neoplasia (VIN) lesion, one representative donor of two shown. Counterstaining was done using haematoxylin. Scale bar, 500 μm. (**f**) IFRD1, RelA K310 acetylation and total RelA levels in 24-h non- or IFN-γ- and TNF-α-stimulated control or IFRD1 knockdown (KD) HPV16+KCs. (**g**) RT–qPCR of *CCL2*, *RANTES*, *IL-8* and *CXCL9* expression in steady-state control or IFRD1 KD HPV16+KCs. (**h**) RT–qPCR of *CCL2*, *RANTES*, *IL-8* and *CXCL9* expression in 24-h non- or IFN-γ- and/or TNF-α-stimulated control or IFRD1 KD HPV16+KCs. (**i**) Enzyme-linked immunosorbent assay for CCL2, RANTES, IL-8 and CXCL9 in cleared supernatants of 24-h non- or IFN-γ- and/or TNF-α-stimulated control or IFRD1 KD HPV16+KCs. (**j**) PBMCs migration towards cleared supernatants of 24-h non- or IFN-γ- and TNF-α-stimulated control or IFRD1 KD HPV16+KCs. A representative example of three different donors is shown. These data are representative for at least three independent experiments. Error bars indicate s.d. *P* values were determined using Welch-corrected unpaired *t*-tests. *P<0.05, **P<0.01 and ***P<0.001.

Acetylation of RelA K310 can be regulated by the lysine acetyl transferases (KAT) PCAF (KAT2B), CREBBP (KAT3A), p300 (KAT3B) and TIP60 (KAT5), as well as the HDACs 1 and 3 (ref. 17). Since our results imply that hrHPV has a mechanism either to deacetylate or impair the acetylation of RelA, we screened our validated microarray data^12^ for genes involved in regulating RelA K310 acetylation. HrHPV did not significantly influence HDAC (*HDAC1* to *11*) or sirtuin (*SIRT1* to *7*) expression (Fig. 2a). The only significantly upregulated gene was the lysine acetyl transferase *CREBBP* (*KAT3A*), confirming previous observations stating that HPV upregulates CREBBP to enhance expression from episomal DNA^21^,^22^. However, as CREBBP acetylates RelA its upregulation cannot explain the observed lower levels of RelA K310 acetylation in hrHPV-infected KCs under steady-state conditions. Interestingly, the microarray data also showed the upregulation of *IFRD1* (Fig. 2b), which previously was shown to complex HDAC1 (ref. 23) and HDAC3 to RelA causing its deacetylation at lysine 310 in the mouse myoblast cell line C2C12 (ref. 24). We hypothesized that it may fulfil a similar role in human KCs. Therefore, reverse transcriptase (RT)–quantitative PCR (qPCR) and western blotting was used to confirm that *IFRD1* gene expression (Fig. 2c, left) and IFRD1 protein levels (Fig. 2d, left) were elevated in two independent HPV16+KC cultures. Knockdown of the polycistronic mRNA of HPV16 by a small interfering RNA (siRNA) against HPV16 E2 in HPV16+KCs resulted in the reduction of HPV16 *E1*, *E2*, *E6* and *E7* expression (Supplementary Fig. 1), *IFRD1* mRNA (Fig. 2c, middle) and IFRD1 protein levels (Fig. 2d, middle), indicating that the augmented IFRD1 levels in hrHPV+KCs are the result of the presence of hrHPV. Reciprocally, when undifferentiated KCs were infected with native HPV16 virions, *IFRD1* mRNA (Fig. 2c, right) and IFRD1 protein (Fig. 2d, right) levels were clearly enhanced after 2 days of infection. Furthermore, immunohistochemistry of HPV-positive vulvar lesions revealed the presence of IFRD1 in the nuclei of cells positive for HPV16 E2 (reflecting HPV-infected cells)^25^, but not in the nuclei of already transformed KCs (identified through p16 staining^25^,^26^ or undifferentiated (E2 and p16 negative) healthy tissue (Fig. 2e).

We then asked whether the hrHPV-induced increased levels of IFRD1 affected RelA K310 acetylation also in human undifferentiated KCs. Indeed, when lentivirus-delivered siRNA against *IFRD1* was used to lower IFRD1 protein expression, a concomitant increase in the steady-state levels of acetylated RelA K310 in HPV16+KCs was seen when compared with control knockdown HPV16+KCs (Fig. 2f; Supplementary Fig. 2a). Furthermore, a small increase in total RelA protein levels was observed. The gain in acetylated RelA K310 translated into a higher basal expression and secretion of cytokines in IFRD1 KD cells (Fig. 2g–i), indicating that IFRD1 is involved in the deregulation of steady-state inflammatory gene expression levels in HPV16+KCs. The dampening effect of *IFRD1* on the NFκB-regulated cytokine expression became even more apparent when the KCs were stimulated with both IFN-γ and TNF-α (Fig. 2h,i). The cytokine levels produced after stimulation were much higher in IFRD1 KD HPV16+KCs than in control KD HPV16+KCs. Moreover, *IFRD1* knockdown augmented the ability of HPV16+KCs to attract PBMCs (Fig. 2j). The main results were recapitulated in HPV18-infected KCs (Supplementary Fig. 3), suggesting that IFRD1 may form a general mechanism exploited by any hrHPV type.

As the effect of IFRD1 occurred directly at the level of RelA, the influence of IFRD1 on the response of HPV16+KCs to poly(I:C) stimulation, previously shown by us to be impaired in hrHPV-infected KCs^8^, was also tested. Knockdown of *IFRD1* resulted in an enhanced expression of *CCL2*, *RANTES*, *IL-8* and *CXCL9* following PRR stimulation with poly(I:C) (Supplementary Fig. 4).

Thus, hrHPV upregulates the expression of *IFRD1* soon after infection, thereby effectively decreasing the basal levels of transcriptionally active RelA and as a consequence the levels of pro-inflammatory cytokines induced via various innate and adaptive immune-mediated NFκB stimulatory pathways.

### EGFR signalling mediates the increased expression of IFRD1

**Figure 3:**
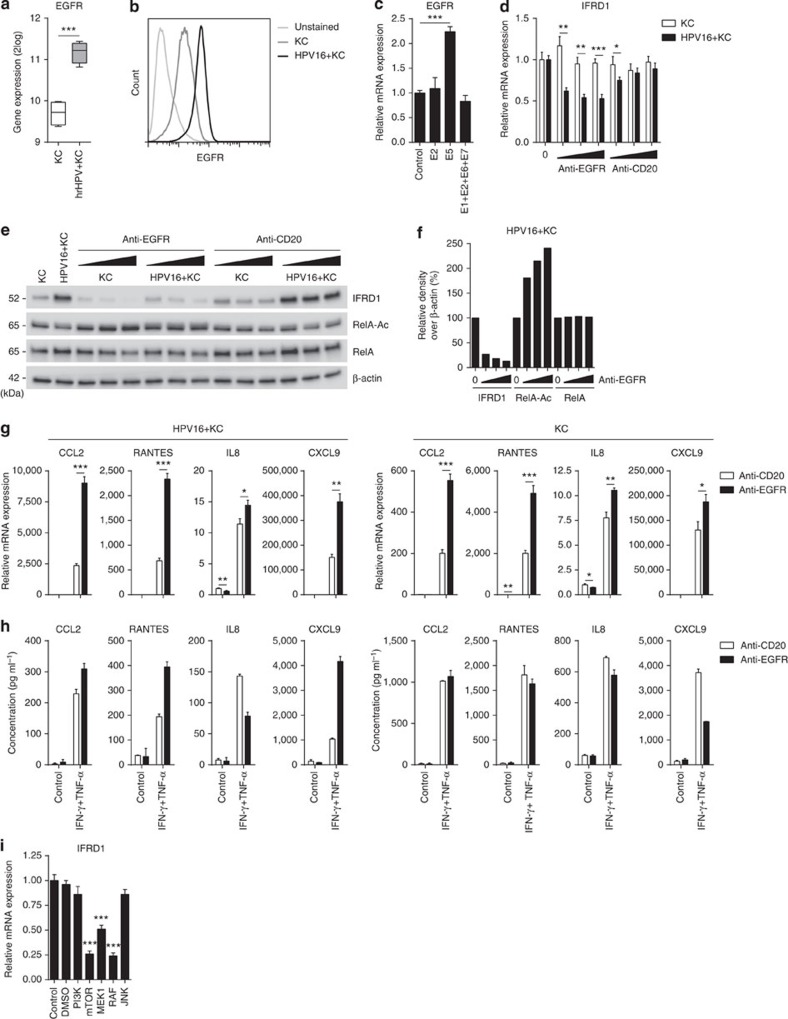
Blocking EGFR signalling decreases IFRD1 levels and rescues cytokine production by hrHPV+KCs. (**a**) Microarray intensities for *EGFR* in KCs (*n*=4) and hrHPV+KCs (*n*=4) represented in a box plot. (**b**) Histogram of EGFR surface protein expression on KCs and HPV16+KCs, as determined by flow cytometry. (**c**) RT–qPCR of *EGFR* expression in KCs transfected with complementary DNA for E2, E5, E1+E2+E6+E7 or empty control. (**d**) RT–qPCR of *IFRD1* expression in KCs and HPV16+KCs treated for 72 h with 0, 0.1, 1 or 10 μg ml^−1^ anti-EGFR or anti-CD20. (**e**) IFRD1, RelA K310 acetylation and total RelA levels in KCs and HPV16+KCs treated for 72 h with 0, 0.1, 1 or 10 μg ml^−1^ anti-EGFR or anti-CD20. (**f**) Quantified protein levels of IFRD1, RelA K310 acetylation and RelA over β-actin in HPV16+KCs treated for 72 h with 0, 0.1, 1 or 10 μg ml^−1^ anti-EGFR (two-dimensional western blot). The expression levels of the 0 μg ml^−1^-treated HPV+KCs were set as 100%. (**g**) RT–qPCR of *CCL2*, *RANTES*, *IL-8* and *CXCL9* expression in 24-h non- or IFN-γ- and TNF-α-stimulated, anti-CD20- or anti-EGFR-treated HPV16+KCs (left) and KCs (right). (**h**) Enzyme-linked immunosorbent assay for CCL2, RANTES, IL-8 and CXCL9 in cleared supernatants of 24-h non- or IFN-γ- and TNF-α-stimulated, anti-CD20- or anti-EGFR-treated HPV16+KCs (left) and KCs (right). (**i**) RT–qPCR of *IFRD1* expression in HPV16+KCs treated with inhibitors of PI3K (LY94002, 25 μM), mTOR (rapamycin, 50 nM), MEK1 (PD98059, 50 μM), RAF (GW5074, 20 μM) and JNK (SP60025, 20 μM). Gene expression was normalized using *GAPDH* as the calibrator gene. Fold changes over control were calculated and depicted. These data are representative for at least three independent experiments, except for **h** that was performed once. Error bars indicate s.d. *P* values were determined using Welch-corrected unpaired *t*-tests. *P<0.05, **P<0.01 and ***P<0.001.

Growth factors, such as nerve growth factor, fibroblast growth factor or EGF, have previously been shown to induce the expression of *Tis* family genes, which also includes *IFRD1*, in rat neocortical astrocytes and chromaffin cell line PC12, mouse C243 and IEC-18, and mammary epithelial cells^27^. The hrHPV E5 protein is known to affect different aspects of EGFR signalling and expression^28^. Verification of EGFR expression in our model showed that *EGFR* mRNA expression (Fig. 3a) and membrane-bound protein expression (Fig. 3b) were higher in hrHPV+KCs than in non-infected KCs. When we transfected complementary DNA for E2 (as control), E5 or a mix of several other HPV proteins, only E5 enhanced *EGFR* expression (Fig. 3c). To test whether EGFR signalling had a similar effect on IFRD1 in human primary KCs, the clinically used anti-EGFR antibody cetuximab was employed to block EGFR signalling. Indeed, *IFRD1* expression decreased in HPV16+KCs, but not in uninfected KCs, when treated with cetuximab (Fig. 3d). IFRD1 protein levels also decreased dose-dependently in both cetuximab-treated non-infected KCs and HPV16+KCs (Fig. 3e). Notably, the isotype control antibody rituximab (anti-CD20) had no effect (Fig. 3d,e). Thus EGFR signalling does not only induce *IFRD1* gene expression but also stabilizes IFRD1 protein levels. Relative density analysis revealed that in cetuximab-treated HPV16+KCs the protein levels of IFRD1 decreased while concomitantly the levels of RelA K310 acetylation increased in a dose-dependent fashion. Total RelA levels were unaffected (Fig. 3f). These results indicated that the HPV-induced expression of IFRD1 is mediated via the EGFR signalling pathway, and implied that cetuximab treatment may enhance the hrHPV+KCs pro-inflammatory cytokine response to immune stimuli. Indeed, upon IFN-γ and TNF-α stimulation cetuximab-treated HPV16+KCs expressed higher levels of indicated cytokine genes than rituximab-treated cells (Fig. 3g), as well as higher levels of secreted cytokines (Fig. 3h). In uninfected KCs, treatment with cetuximab decreased the already low levels of IFRD1 protein, and although this led to increased cytokine gene expression after IFN-γ and TNF-α-stimulation, no additional increase in the already high levels of secreted cytokines was observed (Fig. 3g,h). The absence of cytokine production in cetuximab-treated HPV16+KC and uninfected KCs that were not stimulated with IFN-γ and TNF-α shows that binding of cetuximab to EGFR *per se* does not result in the stimulation of cytokine production (Fig. 3g,h).

As EGFR signalling involves the downstream partners PI3K, mTOR, MEK1, RAF and JNK, we selectively inhibited these proteins using small-molecule inhibitors in HPV16+KCs and observed that selective inhibition of mTOR (rapamycin), MEK1 (PD98059) and RAF (GW5074), but not PI3K (LY94002) or JNK (SP60025), resulted in decreased expression of IFRD1 (Fig. 3i). Thus EGFR-mediated upregulation of IFRD1 is fundamental to the impaired NFκB-induced cytokine response of hrHPV-infected KCs to innate and adaptive immune stimuli.

### HDAC1/3 inhibition stimulates cytokine production

**Figure 4:**
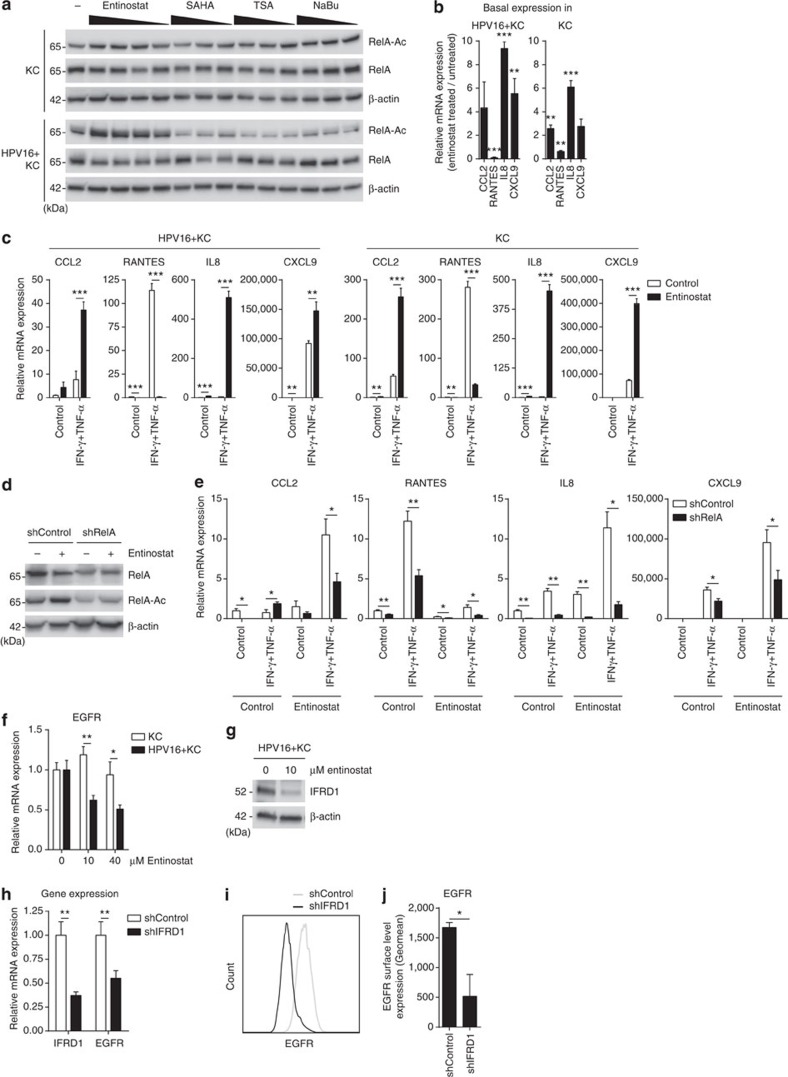
Entinostat treatment reveals involvement of HDAC1/3 in RelA deacetylation in HPV16+KCs. (**a**) RelA K310 acetylation and total RelA levels in KCs and HPV16+KCs treated with decreasing doses of entinostat (40, 20, 10 and 2 μM), SAHA (10, 5 and 1 μM), TSA (5, 1 and 0.333 μM) or NaBu (10, 5 and 1 mM). RT–qPCR of *CCL2*, *RANTES*, *IL-8* and *CXCL9* expression in steady state (**b**) or 24-h non- or IFN-γ- and TNF-α-stimulated (**c**) control or entinostat (10 μM) pre-treated HPV16+KCs. (**d**) Total RelA levels and RelA K310 acetylation in non- or entinostat-treated control or RelA knockdown (KD) HPV16+KCs. (**e**) RT–qPCR of *CCL2*, *RANTES*, *IL-8* and *CXCL9* expression in 24-h non- or IFN-γ- and TNF-α-stimulated non- or entinostat-treated control or RelA knockdown (KD) HPV16+KCs. (**f**) RT–qPCR of *EGFR* expression in KCs and HPV16+KCs treated with increasing doses of entinostat (0, 10 or 40 μM). Gene expression was normalized using *GAPDH* as the calibrator gene. (**g**) IFRD1 in control or entinostat (10 μM) pre-treated HPV16+KCs. (**h**) RT–qPCR of *IFRD1* and *EGFR* expression in control or IFRD1 KD HPV16+KCs. Gene expression was normalized using *GAPDH* as the calibrator gene. Histogram (**i**) and geomean (**j**) of EGFR expression on control or IFRD1 KD HPV16+KCs, as determined by flow cytometry. s.e.m. of two independent experiments. These data are representative for at least two independent experiments. Error bars indicate s.d. *P* values were determined using Welch-corrected unpaired *t*-tests. *P<0.05, **P<0.01 and ***P<0.001.

IFRD1-mediated RelA deacetylation required the recruitment of HDAC1 and/or -3 to the RelA–IFRD1 complex in the mouse myoblast cell line C2C12 (ref. 24). To test whether these HDACs played a similar role in human hrHPV+KCs, the effect of HDAC inhibition was tested in HPV16+KCs and non-infected KCs. A dose titration of the HDAC1/3-specific inhibitor entinostat (MS-275), and the prototypic pan-HDAC inhibitors trichostatin A, sodium butyrate (NaBu) and the Food and Drug Administration-approved vorinostat (suberoylanilide hydroxamic acid (SAHA)) was performed to study RelA K310 acetylation. All pan-HDAC inhibitors increased RelA acetylation in KCs at the lowest concentration used (Fig. 4a; Supplementary Fig. 2b), but at higher doses cells suffered from toxic effects as observed by microscopy. However, HPV16+KCs did survive entinostat treatment, and clearly this HDAC1/3 inhibitor increased RelA K310 acetylation in HPV16+KCs (Fig. 4a; Supplementary Fig. 2b). This indicated that HDAC1 and/or -3 are indeed specifically involved in the deacetylation of RelA in hrHPV+KCs. Entinostat treatment of HPV16+KCs not only restored RelA K310 acetylation but also released the suppressive effect of IFRD1 on cytokine production. Treated HPV16+KCs displayed a higher basal expression for three out of four tested cytokines when compared with their untreated counterparts (Fig. 4b). Moreover, when stimulated with IFN-γ and TNF-α both KCs and HPV16+KCs displayed a higher expression of *CCL2*, *IL-8* and *CXCL9*, although the expression of *RANTES* was abrogated (Fig. 4c). To confirm the involvement of RelA in this process, RelA was knocked down in HPV+KCs (Fig. 4d), after which the cells were treated with entinostat and stimulated with IFN-γ and TNF-α. Indeed, RelA acetylation and cytokine production was increased in the control knockdown cells after stimulation with IFN-γ and TNF-α when treated with entinostat (Fig. 4d,e). However, when RelA was knocked down in HPV16+KCs, the cytokine expression was abrogated despite treatment with entinostat (Fig. 4e).

Previously, it was shown that HDAC inhibition abrogates EGFR expression^29^,^30^, indicating that *EGFR* expression is dependent on acetylation events. Indeed, entinostat treatment dose-dependently abrogated *EGFR* expression in hrHPV+KCs, but did not influence the expression in KCs (Fig. 4f). Furthermore, entinostat treatment resulted in a reduced level of IFRD1 protein in hrHPV+KCs (Fig. 4g), which made us wonder whether IFRD1 could regulate EGFR expression. Therefore, *IFRD1* was knocked down in hrHPV+KCs and this resulted in lower *EGFR* expression (Fig. 4h) and a lower level of membrane-bound EGFR than control-treated hrHPV+KCs (Fig. 4i,j), indicating that IFRD1 can control *EGFR* expression.

### IFRD1 hampers the response of cancer cells to IFN-γ and TNF-α

**Figure 5:**
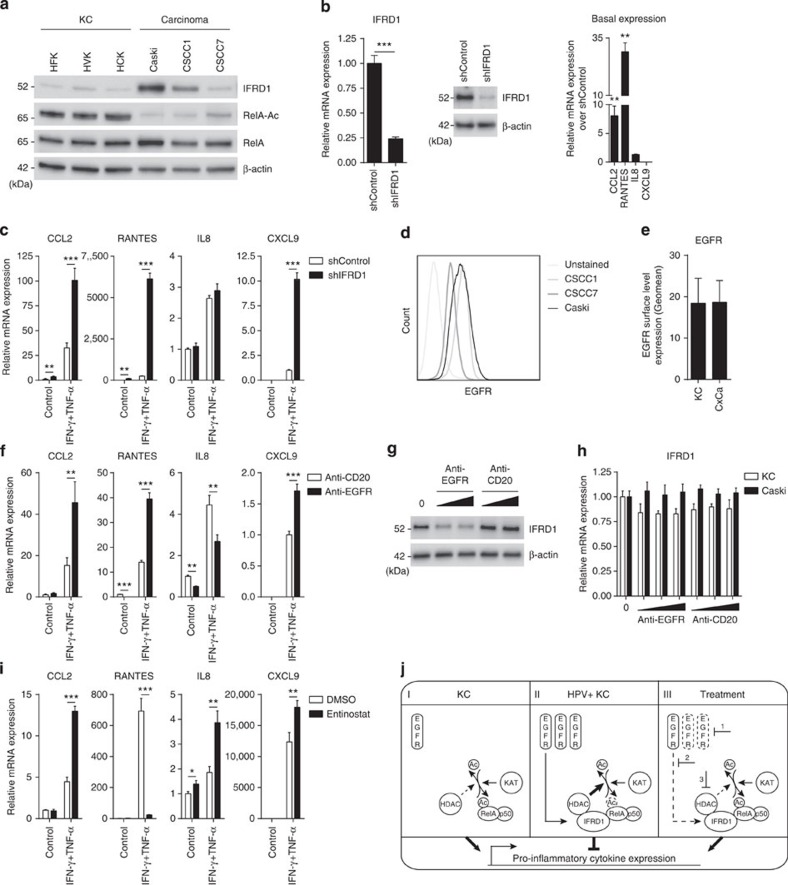
Role of IFRD1 in hrHPV+ cervical cancer cells. (**a**) IFRD1, RelA acetylation and total RelA levels at steady state in three KC donors and three HPV16-induced CxCa lines. (**b**) RT–qPCR of *IFRD1*, *CCL2*, *RANTES*, *IL-8* and *CXCL9* expression, and IFRD1 protein levels in steady-state control or IFRD1 KD Caski cells. (**c**) RT–qPCR of *CCL2*, *RANTES*, *IL-8* and *CXCL9* expression in 24-h non- or IFN-γ- and TNF-α-stimulated control or IFRD1 KD Caski cells. (**d**) Histogram of EGFR expression on three HPV16-induced CxCa lines. (**e**) Geomean of EGFR expression on KCs and CxCa, as determined by flow cytometry. s.e.m. of two independent experiments. (**f**) RT–qPCR of *CCL2*, *RANTES*, *IL-8* and *CXCL9* expression in 24-h non- or IFN-γ- and TNF-α-stimulated anti-CD20- or anti-EGFR-treated Caski cells. (**g**) IFRD1 and RelA K310 acetylation status in Caski cells treated for 72 h with 0, 1 or 10 μg ml^−1^ anti-EGFR (cetuximab) or anti-CD20 (rituximab). (**h**) RT–qPCR of *IFRD1* expression in KCs and Caski cells treated for 72 h with 0, 0.1, 1 or 10 μg ml^−1^ anti-EGFR or anti-CD20. (**i**) RT–qPCR of *CCL2*, *RANTES*, *IL-8* and *CXCL9* expression in 24-h non- or IFN-γ- and TNF-α-stimulated control (dimethylsulphoxide (DMSO)) or entinostat-treated Caski cells. (**j**) Schematic representation of IFRD1-mediated RelA (de-)acetylation. (I) In KCs, RelA acetylation is positively regulated by KATs, resulting in the production of pro-inflammatory cytokines. HDACs may suppress this process. (II) In HPV+KCs, elevated EGFR levels can induce the expression of IFRD1, which can mediate RelA deacetylation by forming a bridge between RelA and HDAC1 and/or -3, hampering pro-inflammatory gene expression. (III) Interfering with EGFR signalling (1 and 2) or HDAC function (3) may lower IFRD1 levels, restoring the RelA acetylation balance, augmenting pro-inflammatory gene expression. Error bars indicate s.d. *P* values were determined using Welch-corrected unpaired *t*-tests. *P<0.05, **P<0.01 and ***P<0.001.

To evaluate whether an increased expression of IFRD1 could also play a role in HPV16-induced squamous cell carcinoma, we analysed the cell line Caski, as well as the two early-passage cervical cancer cell lines, CSCC1 and CSCC7 (ref. 31). IFRD1 protein expression differed between the cell lines (Fig. 5a), but was increased in Caski and CSCC1 when compared with normal KCs. RelA K310 acetylation was lower in all three cervical cancer cell lines than in uninfected KCs (Fig. 5a; Supplementary Fig. 2c). For the Caski and CSCC1 lines this may be explained by the presence of upregulated IFRD1. However, the lack of RelA K310 acetylation in the CSCC7 line indicates that, besides IFRD1 also other mechanisms can alter the acetylation of RelA K310 in these squamous cancer cells.

Because IFRD1 was upregulated in the Caski and CSCC1 cells, we studied the effects of IFRD1 using these cell lines. IFRD1 knockdown in the CSCC1 cells did not alter basal cytokine expression levels (Supplementary Fig. 5a), but IFRD1 knockdown in the Caski cells resulted in a direct increase of the basal expression levels of *CCL2* and *RANTES* (Fig. 5b). Furthermore, both cell lines showed increased cytokine gene levels upon IFN-γ and TNF-α stimulation when IFRD1 was knocked down as compared with their control knockdown counterparts (Fig. 5c; Supplementary Fig. 5b).

CSCC1 and Caski cells express EGFR (Fig. 5d) at a level that is similar to that of uninfected KCs (Fig. 5e). However, the downstream signalling pathway is known to be constitutively higher in HPV-induced cancer cells^32^. As a consequence, the treatment of Caski and CSCC1 cancer cells with the anti-EGFR antibody cetuximab resulted in a higher production of IFN-γ and TNF-α-induced cytokines than when the cancer cells were treated with the control anti-CD20 antibody rituximab (Fig. 5f; Supplementary Fig. 5c). The enhanced response to IFN-γ and TNF-α was associated with a concomitant decrease in IFRD1 protein levels (Fig. 5g), but not mRNA expression (Fig. 5h), upon EGFR blockade. Similarly, treatment of Caski and CSCC1 cancer cells with entinostat resulted in a higher production of *CCL2*, *IL-8* and *CXCL9* by the cancer cells when stimulated with IFN-γ and TNF-α than dimethylsulphoxide carrier control-treated cells (Fig. 5i; Supplementary Fig. 5d). Congruent with our earlier observations, *RANTES* levels diminished after entinostat treatment. These results suggest that IFRD1 may also play a role in suppressing the response of cancer cells to immune stimuli such as IFN-γ and TNF-α.

## Discussion

Using a unique *in vitro* model, we here show that hrHPV infection leads to the upregulated expression of endogenous IFRD1 to deregulate the K310 acetylation of NFκB/RelA. As a result, hrHPV-infected KCs display an impaired production of pro-inflammatory cytokines and chemokines, and a reduced capacity to attract immune cells. The increased expression of IFRD1 in hrHPV+KCs is mediated by EGFR signalling via mTOR, RAF and/or MEK1. Knockdown of *IFRD1* with siRNA or indirectly via blockade of EGFR with the clinically used EGFR-specific antibody cetuximab, resulted in decreased IFRD1 mRNA and protein levels, increased NFκB/RelA K310 acetylation and enhanced expression and production of pro-inflammatory cytokines and chemokines by hrHPV+KCs. The use of entinostat indicated that HDAC1 and/or -3 are involved in lowering K310 acetylation of NFκB/RelA. These conclusions are schematically represented in Fig. 5j.

EGFR activation on epithelial cells has been shown to result in a decreased production of CCL2, RANTES and CXCL10 and increased production of IL-8. Inhibition of EGFR signalling with blocking antibodies or tyrosine kinase inhibitors can reverse the effect on these cytokines, as well as result in an increased epithelial immune infiltrate *in vivo*^33^,^34^,^35^. Interestingly, virus-induced EGFR activation has been implicated as a novel mechanism for respiratory viruses to suppress antiviral host responses^33^. The exact underlying mechanism on EGFR-mediated immune suppression remained unclear, albeit that ERK1/2 signalling was shown to be involved in regulating cytokine production and skin inflammation^36^. Using the EGFR-blocking antibody cetuximab in the absence of an additional EGFR stimulus such as the transforming growth factor-α, we found similar effects on the cytokine production of HPV16+KCs. In KCs, the expression of EGFR and IFRD1 are tightly linked, as EGFR inhibition reduced the expression and protein levels of IFRD1, via mTOR, RAF and/or MEK1, but not PI3K or JNK. This fits with the involvement of ERK1/2 in regulating cytokine production^36^, since RAF and MEK1 are just upstream of these kinases. On the basis of our data, the previously observed EGFR activation-induced suppression of cytokine production and immune cell infiltration of epithelia can be explained by upregulation of IFRD1 and subsequent suppression of NFκB signalling. Our data suggest that EGFR-driven overexpression of IFRD1 may also play a role in deregulating NFκB-signalling in HPV-induced tumour cells. Knockdown of IFRD1 results in an increased production of pro-inflammatory cytokines and chemokines by tumour cells when stimulated with IFN-γ and TNF-α. Furthermore, blocking of the EGFR by cetuximab resulted in a decrease of IFRD1 protein levels, as well as increased cytokine production. The HPV oncoproteins are also known to directly intervene with NFκB signalling. Studies with transfected or transformed cells—resembling protein expression in tumour cells—show that E6 and/or E7 proteins inhibit basal and TNF-α-inducible NFκB activity^37^ by influencing NFκB localization^38^,^39^ and activation^40^,^41^,^42^,^43^.

Studies in immunosuppressed patients and healthy individuals show a key role for the adaptive immune response, in particular that of a strong type-1 (IFN-γ and TNF-α)-associated HPV early antigen-specific T cells in the protection against progressive disease^5^. This notion is sustained by the clinical responses of patients treated with HPV-specific therapeutic vaccines^5^. Ample reasons, therefore, for HPV to also develop strategies preventing KCs to respond to these cytokines. Our data show that HPV deploys multiple strategies to interfere with induced RelA-associated NFκB signalling. HPV utilizes the cellular deubiquitinase UCHL1 to interfere with TRAF3, TRAF6 and NEMO function^8^, and here we show that HPV also upregulates the expression of endogenous IFRD1 to deregulate the K310 acetylation of NFκB. Furthermore, the E7 protein of hrHPV has been shown to bind HDAC1 and prevent acetylation of histones, thereby suppressing TLR9 signaling^44^, but E7 can also displace HDACs resulting in enhanced hypoxia-inducible factor-1α transcriptional activity^45^. It is not unusual for viruses to target NFκB activation^46^,^47^, and hampering RelA acetylation is a common strategy. For instance, the N terminus of the orf virus protein 002 inhibits acetylation of RelA by blocking phosphorylation of RelA S276 and subsequent recruitment of acetylases p300 and CBP^48^, and the A238L protein of the African swine fever virus hampers RelA K310 acetylation by inhibiting RelA–p300 interaction^49^. We here postulate that hrHPV does not hamper KATs in acetylating RelA, but rather recruits a mediator to enhance HDAC-mediated RelA deacetylation. Together with our observation that HPV lowers basal cytokine expression in resting KCs due to the presence of IFRD1, we suggest that impairment of immune-driven RelA-associated NFκB-responsive gene expression is crucial for the virus to persist. This viral strategy has not been reported before, but as discussed above may also be employed by respiratory viruses that activate EGFR^33^.

All together, our data indicate that HPV upregulates EGFR to drive IFRD1 expression as a tool to decrease basal and adaptive-immune system-driven cytokine expression. This may allow hrHPV to evade the host’s immune response. It is highly likely that this mechanism plays a role in other viral infections too and even extends to tumours.

## Methods

### Ethics statement

The use of discarded human foreskin, cervical and vaginal KC tissues to develop cell lines for these studies was approved by the institutional review board at the Pennsylvania State University College of Medicine and by the institutional review board at Pinnacle Health Hospitals. The Medical Ethical Committee of the Leiden University Medical Center approved the human tissue sections (healthy foreskin, healthy cervix and HPV16- or 18-positive cervical neoplasias) used for staining. All sections and cell lines were derived from discarded tissues and de-identified, therefore no informed consent was necessary.

### Cell culture

Primary cultures of human epithelial KCs were established from foreskin, vaginal, vulva and cervical tissues, as previously described^3^, and grown in KC serum-free medium (Medium 154 supplemented with the HKGS kit, Invitrogen, Breda, The Netherlands). KCs stably maintaining the full episomal HPV genome following electroporation (HPV-positive KCs) were grown in monolayer culture using E-medium in the presence of mitomycin C (Sigma-Aldrich, Zwijndrecht, The Netherlands)-treated J2 3T3 feeder cells^19^,^20^ for two passages and were then adapted to KC serum-free medium for one passage before experimentation. J2 3T3 mouse fibroblasts, Caski, CSCC1, CSCC7 and SiHa cell lines were cultured in Iscove’s modified Dulbecco’s medium (IMDM) supplemented with 8% fetal bovine serum, 2 mM l-glutamine and 1% penicillin–streptomycin (complete IMDM medium) (Gibco-BRL, Invitrogen).

### HPV16 infection of non-infected KCs

Primary basal layer human foreskin KCs were seeded at 75,000 cells per well in 24-well plates and allowed to attach for 48 h. Cells received fresh medium (mock infected) or medium containing native HPV16 isolated from raft cultures at multiplicity of infection 100 for 24 h. Cells were washed and harvested for either RT–qPCR or western blotting analysis.

### IFRD1 and RelA knockdown in HPV-positive KCs

shRNAs were obtained from the MISSION TRC-library of Sigma-Aldrich. The MISSION shRNA clones are sequence-verified shRNA lentiviral plasmids (pLKO.1-puro) provided as frozen bacterial glycerol stocks (Luria broth, carbenicillin at 100 μg ml^−1^ and 10% glycerol) in *Escherichia coli* for propagation and downstream purification of the shRNA clones. pLKO.1 contains the puromycin selection marker for transient or stable transfection. The construct against IFRD1 (NM_001550) was TRCN0000156194: 5′-CCGGCAGTTCTGAAACAGTTTCTTTCTCGAGAAAGAAACTGTTTCAGAACTGTTTTT-3′, RelA (NM_021975) was TRCN0000014687: 5′-CCGGCCTGAGGCTATAACTCGCCTACTCGAGTAGGCGAGTTATAGCCTCAGGTTTTT-3′ and the control was: SHC004 (MISSION TRC2-pLKO puro TurboGFP shRNA control vector): 5′-CCGGCGTGATCTTCACCGACAAGATCTCGAGATCTTGTCGGTGAAGATCACGTTTTT-3′. HPV16-positive KCs at ~60% confluence were transduced with lentivirus at multiplicity of infection 5–10 overnight, after which the medium was replaced. At least 72 h post transduction, cells were stimulated as indicated and target gene expression was assayed by RT–qPCR or western blotting.

### HPV knockdown in HPV-positive KCs

Silencer Select siRNA against HPV16 E2 (5′-AACACUACACCCAUAGUACAUtt-3′) was designed using siRNA Target Finder software (Ambion, Invitrogen). Blast search revealed that the designed E2 siRNA does not match with the known human transcriptome. E2 and negative control #2 siRNA (sequence not provided by the manufacturer) were purchased from Ambion. HPV16+KCs were transfected with 50 nM siRNA E2 or negative control #2 using Lipofectamine 2000 (Invitrogen), according to the manufacturer’s instructions. Forty-eight hours post transfection, cells were harvested or stimulated as indicated and target gene expression was assayed by RT–qPCR or western blotting.

### Transfection of HPV genes into non-infected KCs

Non-infected primary KCs were seeded at 50,000 cells per well in 24-well plates and allowed to attach overnight. Cells were transfected with 500 ng DNA using Lipofectamine (Invitrogen), according to the manufacturer’s instructions. Cells were maintained in E-medium. Seventy-two hours post transfection, cells were harvested and target gene expression was assayed by RT–qPCR.

### EGFR signalling blocking

Subconfluent cells were cultured in respective complete growth medium in the presence of cetuximab (0.1, 1 or 10 μg ml^−1^; Merck serono), rituximab (0.1, 1 or 10 μg ml^−1^; Roche), rapamycin (50 nM; Calbiochem), PD98059 (50 μM; Sigma-Aldrich), GW5074 (20 μM; Sigma-Aldrich), LY94002 (25 μM; Sigma-Aldrich) or SP60025 (20 μM; Sigma-Aldrich). Medium was changed every 2–3 days. After at least 72 h, cells were stimulated as indicated and target gene expression was assayed by RT–qPCR or western blotting.

### HDAC inhibition

Subconfluent cells were cultured in presence of a dilution series of entinostat (MS-175; 40, 20, 10 and 2 μM; Selleckchem BioConnect), vorinostat (SAHA; 10, 5 and 1 μM; Sigma-Aldrich), trichostatin A (5, 1 and 0.333 μM; Sigma-Aldrich) or sodium butyrate (NaBu; 10, 5 and 1 mM; Sigma-Aldrich) in the respective complete growth medium overnight. Medium was changed for the respective complete growth medium, cells were stimulated as indicated and target gene expression was assayed by RT–qPCR or western blotting. Since treatment with 10 μM entinostat showed a good increase in RelA K310 acetylation without signs of toxicity, subsequent experiments were performed using this dose.

### Migration assays

(HPV-positive) KCs were stimulated as indicated for 24 h. Cleared (HPV-positive) KC supernatants were added to the lower compartment of a transwell plate (Corning). The upper compartment was filled with PBMCs isolated from buffy coats (Sanquin). PBMCs were allowed to migrate for 16 h, after which the cells in the lower compartment were counted by flow cytometry in the presence of counting beads (Invitrogen), according to the manufacturer’s instructions. Myeloid cells and lymphocytes were differentiated by their respective size in the Forward Scatter (FSC)/Side Scatter (SSC) plot (data not shown).

### RNA expression analyses and ELISA

The microarray data^12^ are accessible in the Gene Expression Omnibus database (accession number GSE54181). Plots were generated using the webtool R2: microarray analysis and visualization platform ( http://r2.amc.nl).

Total RNA was isolated using the NucleoSpin RNA II kit (Machery-Nagel, Leiden, The Netherlands) according to the manufacturer’s instructions. Total RNA (0.5–1.0 μg) was reverse transcribed using the SuperScript III First Strand synthesis system from Invitrogen. TaqMan PCR was performed using the TaqMan Universal PCR Master Mix and pre-designed, pre-optimized primers and probe mix for CCL2, RANTES (CCL5), IL-8 (CXCL8), CXCL9 and GAPDH (Applied Biosystems, Foster City, USA). Threshold cycle numbers (Ct) were determined using the CFX PCR System (Bio-Rad, Veenendaal, The Netherlands), and the relative quantities of complementary DNA per sample were calculated using the ΔΔCt method using GAPDH as the calibrator gene.

Enzyme-linked immunosorbent assays (ELISAs) for CCL2, RANTES, IL-8 and CXCL9 were performed according to the manufacturer’s instruction (PeproTech, London, UK).

### Flow cytometry

Expression of EGFR on KCs was analysed by flow cytometry using phycoerythrin (PE)-coupled mouse-anti-human EGFR (1:20, BD Biosciences, Breda, The Netherlands). Per live gate, 50,000 cells were recorded using the BD FACS Calibur with Cellquest software (BD Bioscience) and data were analysed using Flowjo (Treestar, Olten, Switzerland).

### Western blot analysis

For western blotting, polypeptides were resolved by SDS–polyacrylamide gel electrophoresis and transferred to a nitrocellulose membrane (Bio-Rad). Immunodetection was achieved with anti-p65 (1:1,000, sc-372, Santa Cruz), anti-phospho-p65 (Ser536; 1:1,000, #3033 Cell Signaling Technology (CST)), anti-acetyl-p65 (Lys310; 1:1,000, #3045 CST), anti-IFRD1 (1:400, T2576 Sigma-Aldrich), β-actin (1:10,000, Sigma-Aldrich) primary antibodies, and horseradish peroxidase-coupled anti-mouse (1:5,000; CST) and horseradish peroxidase-coupled anti-rabbit (1:5,000, CST) secondary antibodies. Chemoluminescence reagent (Bio-Rad) was used as a substrate and the signal was scanned using the Chemidoc and accompanying software (Bio-Rad) to quantify the intensity of the bands as a measure of the amount of protein of interest in the blot. The relative amount was determined by calculating the ratio of each protein over that of the density measured for the housekeeping protein β-actin. Images have been cropped for presentation. Full-size images are presented in Supplementary Fig. 6.

### Immunohistochemistry

Four μm formalin-fixed, paraffin-embedded tissue sections from two random vulvar intraepithelial neoplasia cases were deparaffinized and rehydrated using graded concentrations of ethanol to distilled water. Endogenous peroxidise activity was blocked with 0.03% H_2_O_2_/MeOH for 20 min. Antigen retrieval was performed in boiling EDTA buffer (pH 9.0) for 12 min. After 2 h of cooling down to room temperature, slides were washed twice in distilled water and twice in PBS. Subsequently, incubation was performed overnight at room temperature with the primary IFRD1 antibody (T2576 Sigma-Aldrich; 1:500 in PBS containing 1% bovine serum albumin); p16 (CINTEC, diluted 1:5) and E2 (1:50) (provided by Dr F. Thierry). Second, sections were incubated with BrightVision polyhorseradish peroxidase anti-mouse/rabbit/rat immunoglobulin-G (Immunologic BV, Duiven, The Netherlands) for 30 min at room temperature. Washing between incubations was performed three times for 5 min in PBS. Immune complexes were visualized by applying a 0.05-M tris-HCl buffer (pH 7.6) containing 0.05% of 3,3′-diamino-benzidine-tetrahydrochloride and 0.0018% of H_2_O_2_. After 10 min, the reaction was stopped by rinsing with demineralized water. Finally, the tissue sections were counterstained with Mayer’s haematoxylin before addition of a coverslip.

### Statistical analysis

Statistical analysis was performed using GraphPad InStat version 3.00. *P* values were determined using Welch-corrected unpaired *t*-tests. *P<0.05, **P<0.01 and ***P<0.001.

## Additional information

**How to cite this article:** Tummers, B. *et al.* The interferon-related developmental regulator 1 is used by human papillomavirus to suppress NFκB activation. *Nat. Commun.* 6:6537 doi: 10.1038/ncomms7537 (2015).

## Supplementary Material

Supplementary Information Supplementary Figures 1-6
