# Supplementary material for: The interferon-related developmental regulator 1 is used by human papillomavirus to suppress NFκB activation
Source: Nat Commun. 2015 Mar 13;6:6537. doi: 10.1038/ncomms7537 (PMC4382698; doi:10.1038/ncomms7537)
Supplement: Supplementary Information — Supplementary Figures 1-6 [file ncomms7537-s1.pdf]

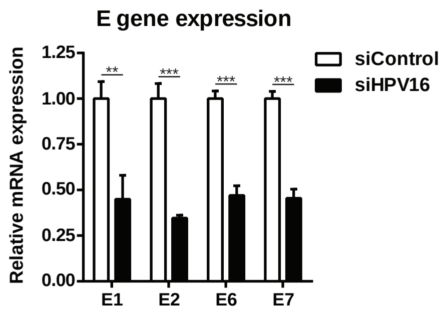

Supplementary Figure 1: HPV16 E gene expression after HPV16 knock-down in HPV16+ KCs
E1, E2, E6 and E7 expression in HFK16 cells transfected with siControl or siHPV16.
Error bars indicate SD. P-values were determined via Welch-corrected unpaired *t* tests. ** $p<0.01$, *** $p<0.001$

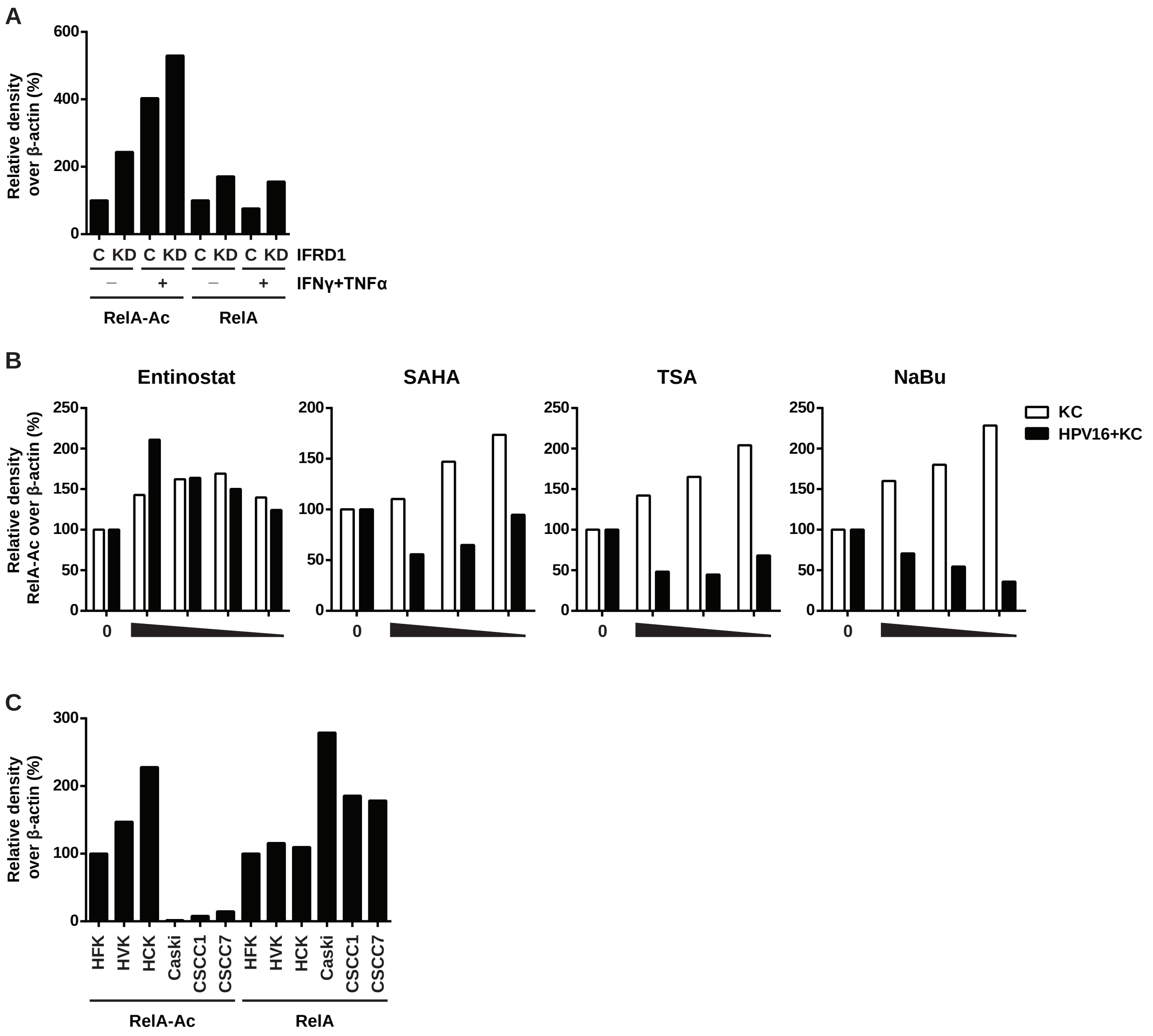

Supplementary Figure 2: Western blot quantifications of RelA acetylation

(**A**) Quantified protein levels of RelA K310 acetylation and RelA over β-Actin in 24 hours non- or IFNγ and TNFα-stimulated control or IFRD1 knock-down (KD) HPV16+ KCs. The expression levels of the control-treated HPV16+ KCs were set as 100%.

(**B**) Quantified protein levels of RelA K310 acetylation over β-Actin in KCs and HPV16+ KCs treated with decreasing doses of entinostat (40, 20, 10 and 2 μM), SAHA (10, 5 and 1 μM), TSA (5, 1 and 0.333 μM) or NaBu (10, 5 and 1 mM) (western blot Figure 4A). The expression levels of the control-treated HPV16+ KCs were set as 100%.

(**C**) Quantified protein levels of RelA K310 acetylation and RelA over β-Actin in in three KC donors and three HPV16-induced CxCa lines. The expression levels of the HFK were set as 100%.

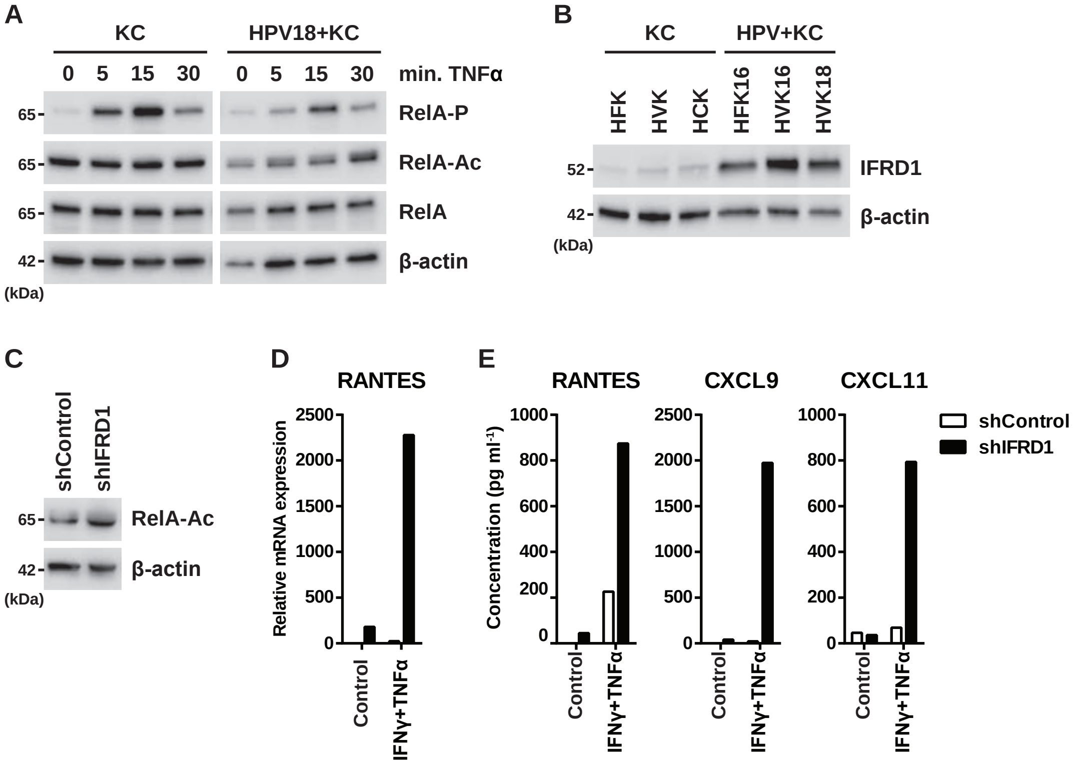

Supplementary Figure 3: RelA acetylation, IFRD1 expression and IFRD1 knock-down effects in HPV18+ KCs

(**A**) RelA phosphorylation, acetylation and total levels in KCs and HPV18+ KCs stimulated with TNFα for 0, 5, 15 and 30 minutes.
(**B**) IFRD1 levels in three KC donor pools, two HPV16+ KC lines and one HPV18+ KC line.
(**C**) RelA acetylation levels in control or IFRD1 knock-down HPV18+ KCs.
(**D**) RT-qPCR of RANTES expression in 24 hours non- or IFNγ and TNFα-stimulated control or IFRD1 knock-down HPV18+ KCs.
(**E**) ELISA for RANTES, CXCL9 and CXCL11 in cleared supernatants of 24 hours non- or IFNγ and TNFα-stimulated control or IFRD1 knock-down HPV18+ KCs.

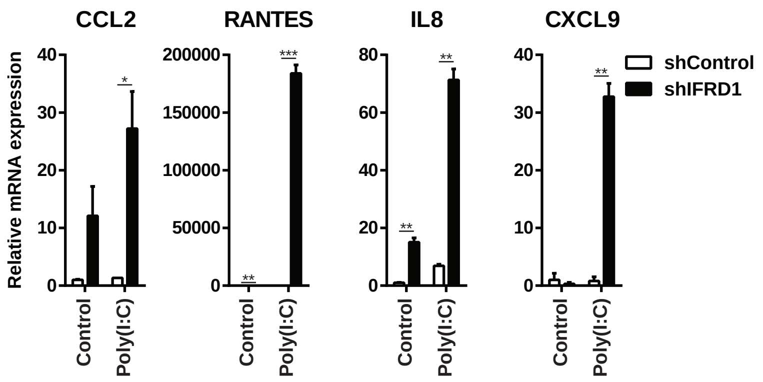

Supplementary Figure 4: IFRD1 impairs Poly(I:C)-induced cytokine expression
RT-qPCR of *CCL2*, *RANTES*, *IL8* and *CXCL9* expression in 24 hours non- or Poly(I:C)-stimulated control or IFRD1 knock-down HPV16+ KCs. These data are representative for at least two independent experiments. Error bars indicate SD.
P-values were determined via Welch-corrected unpaired *t* tests. * $p<0.05$, ** $p<0.01$, *** $p<0.001$.

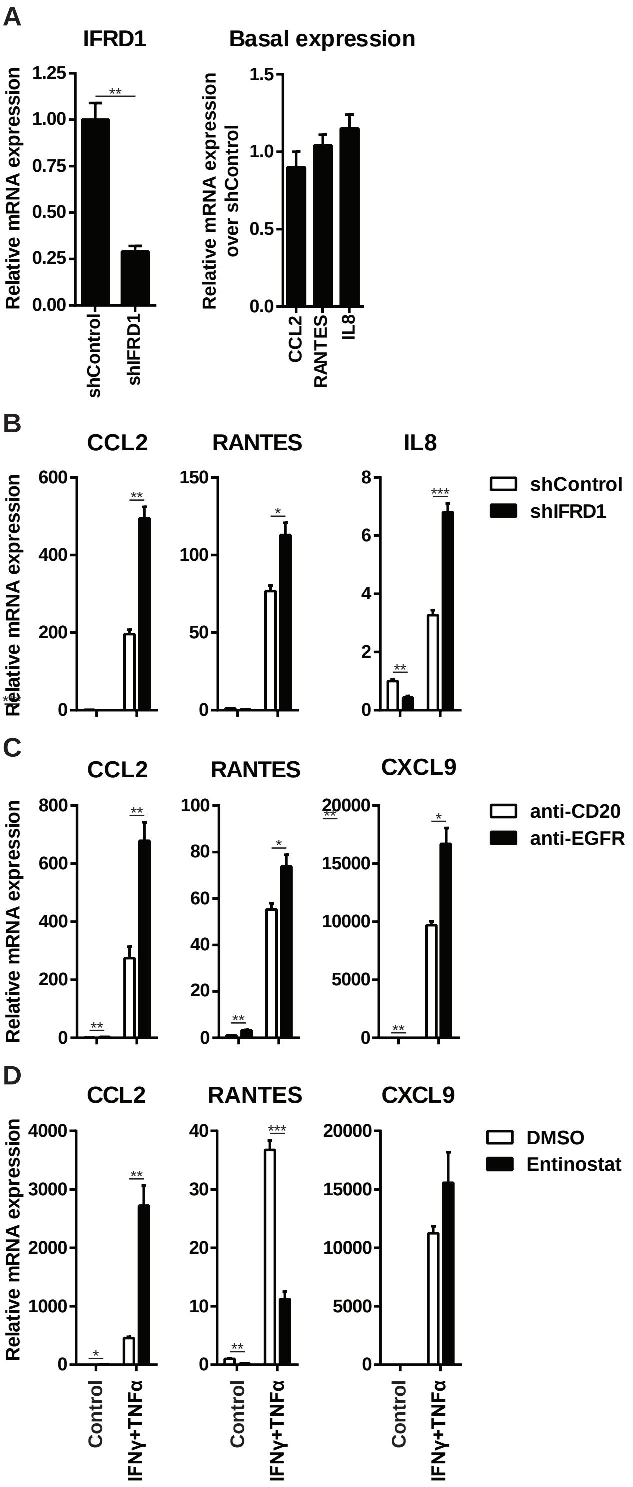

Supplementary Figure 5: The effects of IFRD1 knock-down, anti-EGFR and entinostat on CSCC1
(**A**) RT-qPCR of IFRD1, CCL2, RANTES, IL8 and CXCL9 expression in steady-state control or IFRD1 knock-down CSCC1 cells.
(**B**) RT-qPCR of CCL2, RANTES and IL8 expression in 24 hours non- or IFNγ and TNFα-stimulated control or IFRD1 knock-down CSCC1 cells.
(**C**) RT-qPCR of CCL2, RANTES and CXCL9 expression in 24 hours non- or IFNγ and TNFα-stimulated anti-CD20 or anti-EGFR-treated CSCC1 cells.
(**D**) RT-qPCR of CCL2, RANTES and CXCL9 expression in 24 hours non- or IFNγ and TNFα-stimulated DMSO (control) or entinostat-treated CSCC1 cells.
These data are representative for at least two independent experiments. Error bars indicate SD. P-values were determined via Welch-corrected unpaired *t* tests.
* p<0.05, ** p<0.01, *** p<0.001.

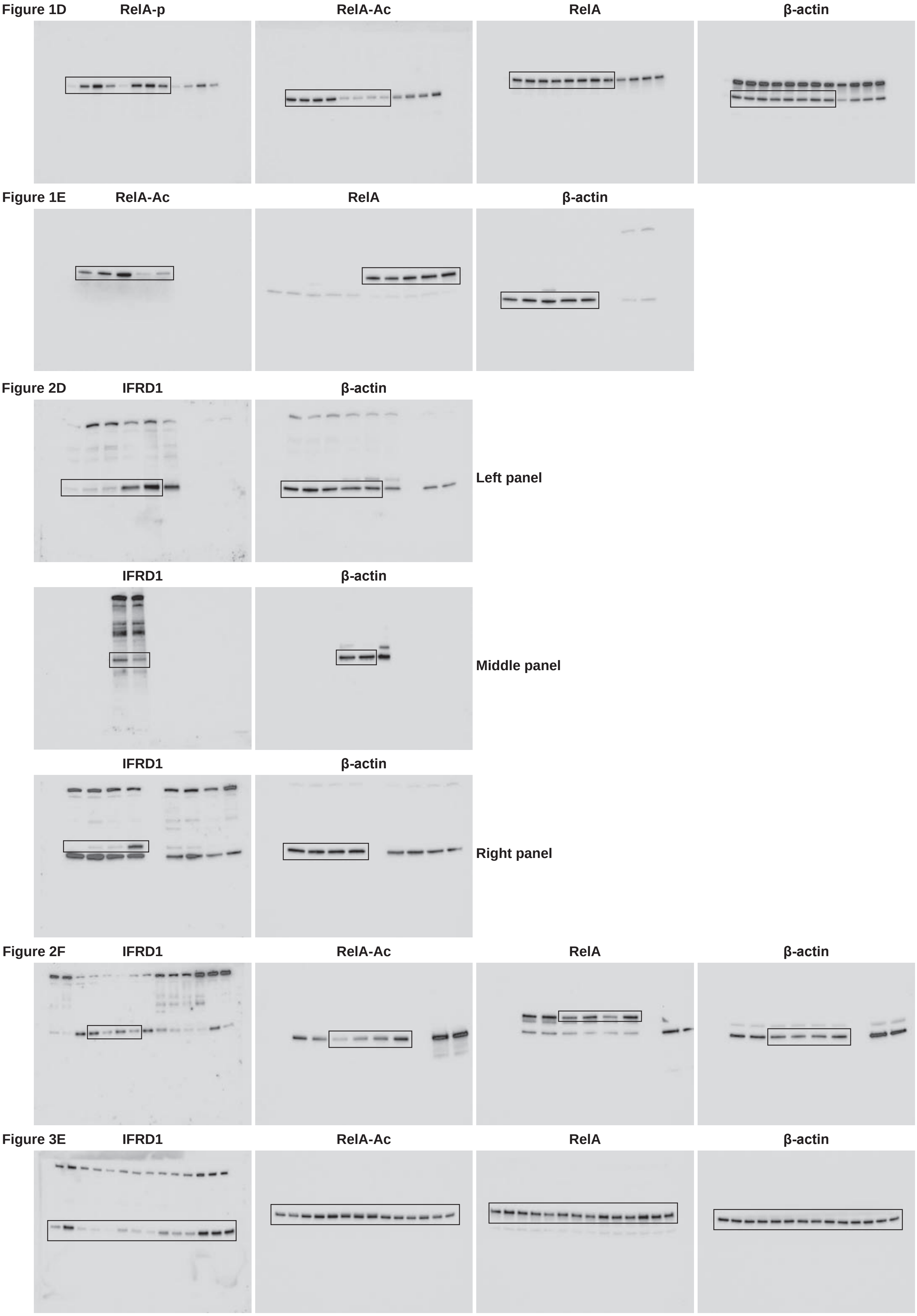

Supplementary Figure 6: Full Western Blot data

The full blots for all Western blot pictures. Above the blot the used target is indicated. The black boxes represent the depicted parts of the blot.

Figure 4A

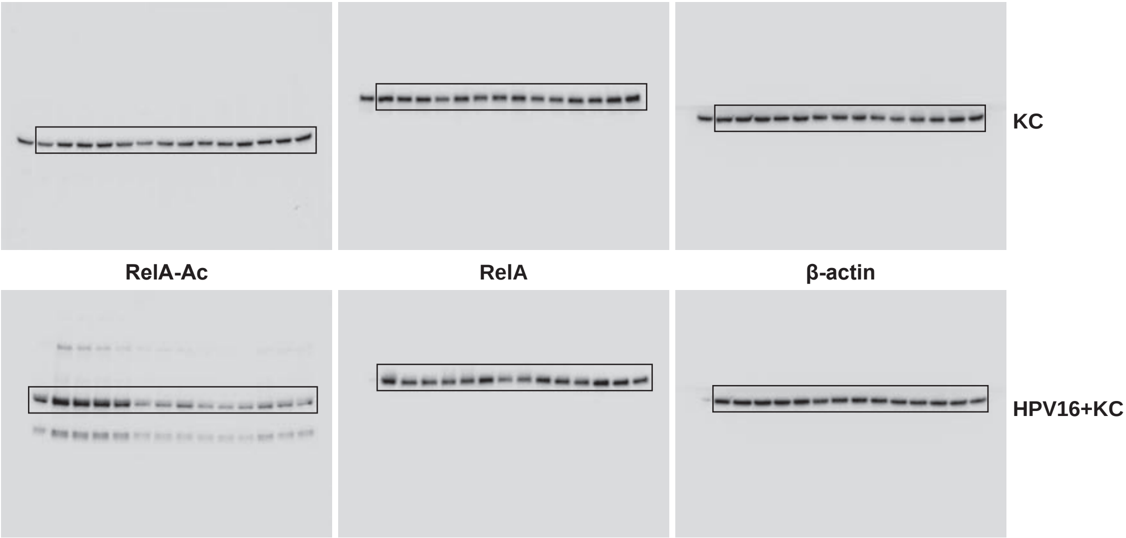

Figure 4D

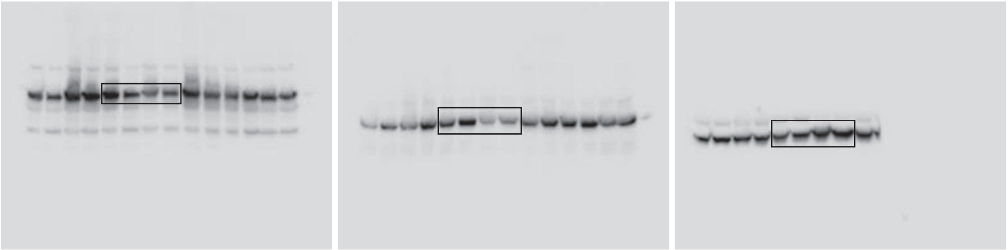

Figure 4G

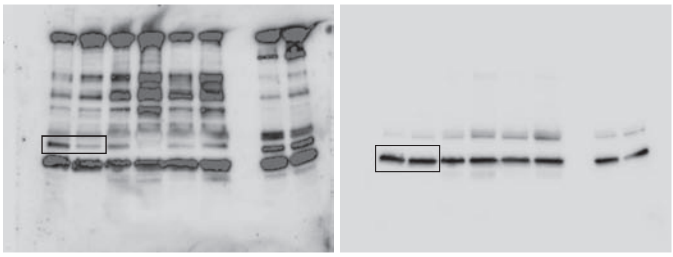

Figure 5A

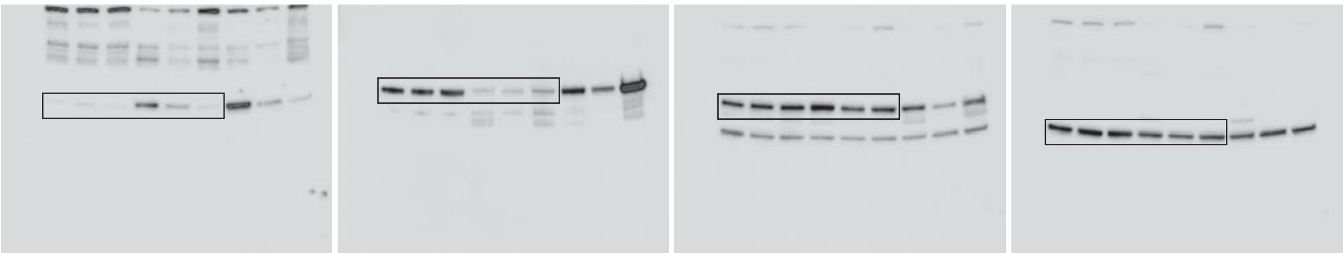

Figure 5B

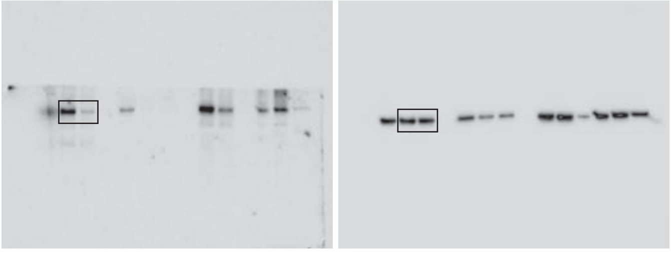

Figure 5G

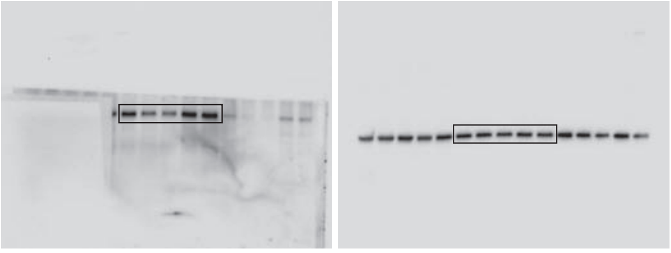

Supplementary Figure 6: Full Western Blot data *Continued*

Supplementary Figure 3A

RelA-p **RelA-Ac** **RelA** **β-actin**

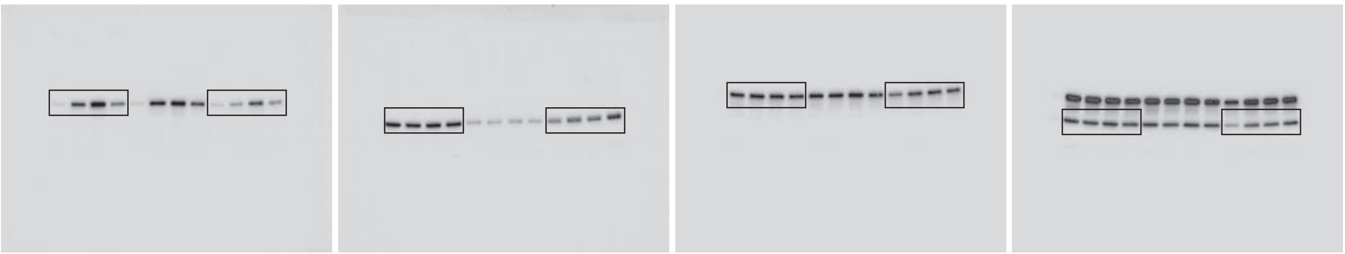

Supplementary Figure 3B

IFRD1 **β-actin**

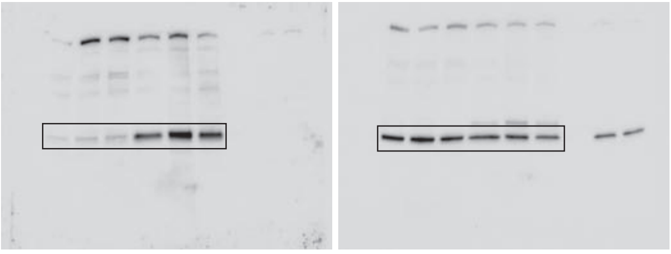

Supplementary Figure 3C

RelA-Ac **β-actin**

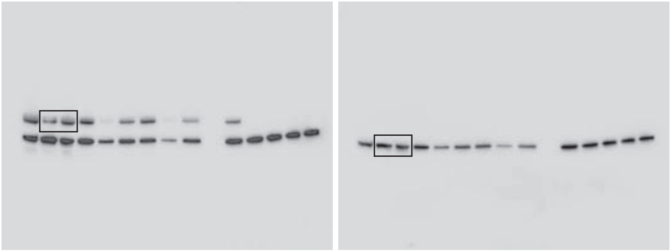

Supplementary Figure 6: Full Western Blot data *Continued*
